# Identifying the Key Mitochondria‐Related Genes in COPD by Integrating Machine Learning and Bioinformatics Analyses

**DOI:** 10.1155/ijog/7060748

**Published:** 2025-10-21

**Authors:** Jiajia Qu, Mengyu Zhang, Yajie Hu, Guang Yang, Xiaoning Zhang, Wenqing Zhang, Yiqing Qu

**Affiliations:** ^1^ Department of Pulmonary and Critical Care Medicine, Qilu Hospital of Shandong University, Jinan, China, qiluhospital.com

**Keywords:** BAX, chronic obstructive pulmonary disease, computational biology, DLST, mitochondrial diseases

## Abstract

**Background:**

Chronic obstructive pulmonary disease (COPD), a prevalent chronic respiratory disorder with high morbidity and mortality, is closely associated with mitochondrial dysfunction and immune dysregulation; however, the underlying mechanisms remain unclear.

**Aims:**

The aim of this study is to identify mitochondrial hub genes and evaluate their diagnostic potential in COPD.

**Methods:**

This study combined bioinformatics and experimental methods to investigate mitochondria‐related differentially expressed genes (MitoDEGs) in COPD pathogenesis. Two GEO datasets (GSE38974/GSE8545) were analyzed to identify MitoDEGs, which were functionally characterized and refined via machine learning (LASSO/SVM‐RFE). Key genes were further validated using quantitative real‐time polymerase chain reaction (qRT‐PCR) and Western blot in bronchial epithelial cells and COPD mouse model lung tissues. Immune infiltration analysis revealed connections between MitoDEGs and immune dysregulation in COPD that were experimentally confirmed using immunohistochemistry (IHC) and immunofluorescence (IF).

**Results:**

Among 28 identified MitoDEGs, five feature genes (*BAX*, *DLST*, *FKBP10*, *FUNDC2*, and *RMDN1*) were selected. Subsequent validation through qRT‐PCR, Western blot, and ROC curve further confirmed *BAX* and *DLST* as core hub genes. Immune profiling revealed significantly increased M0 macrophage infiltration and reduced activated NK cells in COPD. BAX and DLST expression was positively correlated with M0 macrophages but negatively with activated NK cells, a finding corroborated by IHC and IF assays.

**Conclusions:**

These findings highlight *BAX* and *DLST* as potential mitochondrial dysfunction biomarkers in COPD, linking their roles to immune cell infiltration. This study provides novel insights into cigarette smoke‐induced COPD pathogenesis and underscores the diagnostic utility of targeting mitochondrial–immune interactions.

## 1. Introduction

Chronic obstructive pulmonary disease (COPD) refers to a common chronic disorder characterized by persistent respiratory symptoms and airflow restriction. It is universally acknowledged that COPD is the third leading mortality factor and it serves as a major determinant of worldwide disability rates [1]. As the global population ages, the COPD‐related healthcare burden is projected to increase significantly [2]. In addition, smoking stands out as the most prominent external risk factor for the onset and progression of COPD [3]. Cigarette smoke (CS) contains numerous harmful substances that induce oxidative stress in airway epithelial cells, thereby resulting in chronic airway inflammation and also disrupting the protease–antiprotease balance. Recent studies suggest that CS‐mediated mitochondrial perturbation triggers p53‐dependent apoptotic pathways and metabolic reprogramming that exacerbate epithelial injury, yet the precise molecular links between mitochondrial stress and immune infiltration in COPD remain largely undefined [4, 5].

Mitochondria, often referred to as the “powerhouse” of the cell, are organelles in eukaryotic cells that generate energy essential for normal cellular function [6]. Beyond energy production, mitochondria play crucial roles in cell differentiation, communication, apoptosis, and regulation of cell growth and the cell cycle [7]. Therefore, maintaining mitochondrial function and homeostasis is indispensable for cellular health and survival. Studies have identified mitochondrial dysfunction as a key factor in the pathogenesis of COPD, although the precise mechanisms remain elusive [8, 9].

Recent advancements in bioinformatics and high‐throughput sequencing technologies have significantly enhanced biomedical research. In particular, machine learning algorithms have emerged as powerful tools for identifying diagnostic genes and functional molecules across various diseases. Despite these advancements, there still exist several limitations in the application of machine learning when it comes to COPD‐related bioinformatics studies. To address this gap, this study analyzed COPD lung tissue datasets from the Gene Expression Omnibus (GEO) database and utilized a combination of bioinformatics and machine learning techniques to identify key biomarkers, and the results of the analysis were further verified through experimental assays.

## 2. Materials and Methods

### 2.1. Data Collection

Expression profiles from human lung tissue were obtained from NCBI GEO on March 15, 2024. GSE38974 (GPL433; 23 COPD, nine normal) and GSE8545 (GPL570; 15 COPD, 24 normal) served as discovery and validation cohorts, respectively. GSE38974 raw Agilent data were preprocessed in R (limma v3.54.0): background corrected (backgroundCorrect()), quantile normalized (normalizeBetweenArrays()), probes below detection threshold filtered, and gene symbols averaged for multiple probes. GSE8545 CEL files were RMA‐normalized (affy v1.74.0). To minimize technical variation, batch effects across datasets were adjusted using the “ComBat” function in the sva R package (v3.40.0), and expression values were normalized by log2 transformation and quantile normalization. Mitochondrial genes (*n* = 1136) were sourced from MitoCarta3.0. All procedures using human data were exempt from additional institutional review and did not involve direct experimentation with human participants, as both GSE38974 and GSE8545 represent deidentified, publicly available datasets.

### 2.2. Identification of Differentially Expressed Genes (DEGs) and Mitochondria‐Related Differentially Expressed Genes (MitoDEGs)

Normalized GSE38974 data were analyzed using limma. A design matrix contrasted COPD versus controls, fitted with lmFit, and moderated via eBayes. DEGs were defined as |log2FC| > 1 and adj.*p* < 0.05 (Benjamini–Hochberg), yielding 896 DEGs (487 up and 409 down). Intersection with mitochondrial genes identified 28 MitoDEGs (visualized via online Venn diagram tool).

### 2.3. Functional Enrichment Analysis of MitoDEGs

GO (BP [biological process], CC [cellular component], and MF [molecular function]) and Kyoto Encyclopedia of Genes and Genomes (KEGG) enrichment for the 28 MitoDEGs were performed using clusterProfiler (v4.4.1), and *p* values were adjusted for multiple testing using the Benjamini–Hochberg false discovery rate (FDR) method. Terms with adj.*p* < 0.05 were significant. Results were visualized via ggplot2 (v3.4.1) and clusterProfiler.

### 2.4. Identification of Feature Genes

Feature MitoDEGs were selected using least absolute shrinkage and selection operator (LASSO) regression (glmnet v4.1‐5; binomial, *α* = 1; *λ* optimal via 10‐fold CV) and support vector machine–recursive feature elimination (SVM‐RFE) (caret v6.0‐93, e1071 v1.7‐9; RBF kernel; 10‐fold CV for accuracy). Genes selected by both methods (*BAX*, *DLST*, *FKBP10*, *FUNDC2*, and *RMDN1*) were validated in GSE8545 using receiver operating characteristic (ROC) analysis (area under the curve [AUC] of ROC > 0.7 indicated good diagnostic efficiency).

### 2.5. Evaluation of Immune Cell Infiltration

Normalized GSE38974 expression data were submitted to the CIBERSORT web portal (accessed April 5, 2024) using the LM22 signature matrix and 1000 permutations. Samples with CIBERSORT *p* < 0.05 (*n* = 29) were retained, and the estimated proportions of 22 immune cell types were extracted. Spearman′s correlation between each hub gene (*BAX* and *DLST*) and immune cell fraction was calculated in R. Correlation heatmaps and lollipop plots illustrating significant associations (*p* < 0.05) were generated using pheatmap (v.1.0.12) and ggplot2.

### 2.6. Preparation of Cigarette Smoke Extract (CSE)

Each cigarette (Taishan Red General, 11 mg tar content and 1.1 mg nicotine content) was simulated by a peristaltic pump in a 10 mL serum‐free DMEM or RPMI‐1640 medium. They were then titrated to pH 7.35–7.45 with 1 mol/L of sodium hydroxide and sterilized with a 0.22 *μ*m filter for 100% CSE. Serum‐free media diluted the CSE to 0%–7% concentrations, which should be used within 30 min. Quality control was conducted as described by Benedikter et al. The OD of 100% CSE was determined at 320 and 540 nm wavelengths using a microplate reader. If the *Δ*OD (A320‐A540) is between 0.9 and 1.2, the quality of the CSE is satisfactory.

### 2.7. Cell Culture

The Beas‐2B cell line and 16HBE cell line, both human‐derived bronchial epithelial cell lines (Pricella Biotechnology, Wuhan, China), were maintained in DMEM and RPMI‐1640 media, respectively. Both media were enriched with 10% fetal bovine serum and cultured in a humidified 5% CO_2_ atmosphere at 37°C.

### 2.8. RNA Extraction and Quantitative Real‐Time Polymerase Chain Reaction (qRT‐PCR)

Total RNA was extracted from bronchial epithelial cells using the SPARKeasy Cell RNA Kit (SparkJade Science Co., Shandong, China). cDNA was then prepared through premixing Evo M‐MLV RT Premix (Accurate Biotechnology, Hunan, China). The specific primers for human *GAPDH*, *BAX*, and *DLST* (Sangon Biotech, Shanghai, China) are detailed in Table 1. According to the manufacturer′s protocol, qRT‐PCR was performed using the BioRadar PCR CFX‐Connect Real‐Time System and the SYBR Green Premix Pro Taq HS qPCR Kit (Accurate Biotechnology, Hunan, China).

**Table 1:** Primers used for qRT‐PCR in this study.

**Gene**	**Primer sequence (5** ^′^ **-3** ^′^ **)**	**Annealing temperature (°C)**	**Amplification efficiency**
BAX	F: AAGCGACTGATGTCCCTGTCTC	60	1.95
	R: TCTTCTTCCAGATGGTGAGTGAGG		
DLST	F: CAGCAAATGGCGTGATTGAAGC	58	1.98
	R: AGACCTTGACCACCAGGAGAAC		
GAPDH	F: ACAACAGCCTCAAGATCATCAG	60	2.01
	R: GGTCCACCACTGACACGTTG		

### 2.9. Animal Model

Male C57BL/6J mice with a specific pathogen free (SPF) grade of 7 weeks were purchased from GemPharmatech, Jiangsu, China. All mice passed the quality inspection and obtained the quality certificate of experimental animals in Zhejiang Province. A mouse model of emphysema was established through exposure to CS, as described by Pauwels et al. [10]. The mice were randomly divided into two groups: the healthy control group, consisting of 10 mice exposed to normal air, and the emphysema group, consisting of 30 mice placed in a 60 × 45 × 37 cm smoking box. The box had a removable open‐lid roof with a round hole (1 cm in diameter) for every 100 cm and a round hole on the side wall for a cigarette holder. Ten cigarettes were burned in a smoking box each day for approximately 5 min, 5 days a week, over a period of 6 months. The cigarettes used in emphysema models and in vitro production of CSE were Taishan Red General, containing 11 mg of tar and 1.1 mg of nicotine. For in vivo experiments, the study was approved by the Ethics Review Committee of Qilu Hospital, Shandong University (Approval Number: KYLL‐2022(ZM)‐1062). All experimental procedures followed institutional and international ethical standards.

### 2.10. Western Blot Analysis

Mouse lung tissues and cell samples were homogenized in ice‐cold RIPA lysis buffer (Solarbio Technology, Beijing, China) with protease inhibitors (Beyotime, Shanghai, China) for 30 min. Protein quantification was performed using a BCA assay kit (Beyotime, Shanghai, China), followed by the same protein loading on SDS‐PAGE gels after loading buffer normalization (Beyotime, Shanghai, China). Post‐electrophoresis, the protein was transferred to PVDF membranes (Millipore, Bedford, MA, United States). The membranes were then diluted in an antibody buffer (BAX: Abmart, Shanghai, China, 1:2000; DLST: Boster, Wuhan, China, 1:1000; GAPDH: Proteintech, Wuhan, China, 1:5000) and incubated overnight at 4°C. On the following day, a second antibody membrane bound to horseradish peroxidase (HRP) was incubated at room temperature for 1 h: anti‐rabbit IgG (Proteintech, Wuhan, China, 1:10,000) and anti‐mouse IgG (Proteintech, Wuhan, China, 1:10,000). Protein signals were detected using an ultrasensitive enhanced chemiluminescence (ECL) reagent (Yeasen Biotechnology, Shanghai, China), and images of these bands were obtained using a Tanon fully automated chemiluminescence image analysis system. ImageJ software was used to conduct quantitative analysis of the collected images.

### 2.11. H&E Staining

Mice lung tissues were fixed in 4% paraformaldehyde/polyformaldehyde, paraffin‐embedded, and sectioned at a thickness of 4 *μ*m. Sections were deparaffinized in xylene, rehydrated through a graded ethanol series, and rinsed in distilled water. Hematoxylin staining (Servicebio, Wuhan, China) was performed to visualize nuclei, followed by differentiation and bluing (Servicebio, Wuhan, China) and counterstaining with eosin (Servicebio, Wuhan, China) to label cytoplasmic and extracellular matrix components. After dehydration through graded ethanol and clearance in xylene, sections were mounted with neutral resin (Beyotime, Shanghai). Images were captured under a light microscope (Olympus, Munich, Germany) at both low and high magnifications. The mean linear intercept (MLI) and destructive index (DI) were calculated to quantitatively assess alveolar size and the extent of structural destruction, respectively; three sections per mouse and five nonoverlapping fields per section were analyzed.

### 2.12. Immunohistochemistry (IHC)

Paraffin‐embedded lung sections (4 *μ*m) were deparaffinized, rehydrated, and subjected to antigen retrieval in citrate buffer (pH 6.0). Endogenous peroxidase was blocked with 3% H_2_O_2_, and sections were incubated with 5% normal serum. Primary antibodies against F4/80 (Invitrogen, Carlsbad, CA, United States), Ly6C (BioLegend, San Diego, CA, United States), CD8a (Abways, Shanghai, China), and CD206 (Abways, Shanghai, China) were all diluted 1:100–1:200 and applied overnight at 4°C. After PBS washes, HRP‐conjugated secondary antibodies were added and visualized with DAB. Nuclei were counterstained with hematoxylin. Images were captured under a light microscope, and the positive staining area was quantified in five random fields per animal. The average value for each animal was calculated and used as a single biological replicate for statistical analysis.

### 2.13. Immunofluorescence (IF)

Multiplex IF was performed on paraffin‐embedded lung sections using a tyramide signal amplification (TSA) kit (Servicebio, Wuhan, China) following the manufacturer′s instructions. Primary antibodies included BAX (Abmart, Shanghai, China, 1:200), DLST (Boster, Wuhan, China, 1:200), F4/80 (Invitrogen, Carlsbad, CA, United States, 1:200), NKp46 (Proteintech, Wuhan, China, 1:100), and GranzymeB (Proteintech, Wuhan, China, 1:100). After sequential TSA and DAPI counterstaining, images were acquired by a 3DHISTECH scanner under identical settings. The degree of colocalization of epithelial BAX/DLST and immune cells was quantified by calculating Pearson′s correlation coefficient (PCC) using the ImageJ software with the Coloc 2 plugin.

### 2.14. Statistics Analysis

The data were expressed as the mean ± SEM. Statistical analysis was performed using R4.3.2 and IBM SPSS Statistics 26.0. Student′s *t*‐tests were used for comparison between the two separate groups, while Spearman′s correlation was employed for correlation analysis. *p* < 0.05 was considered statistically significant.

## 3. Results

### 3.1. DEGs and MitoDEGs in COPD

Analysis of the GSE38974 dataset under the criteria |log2FC| > 1 and adj.*p* < 0.05 revealed 896 DEGs, comprising 409 downregulated and 487 upregulated genes (Figure 1a,b). Intersection analysis of these DEGs with 1136 mitochondria‐related genes from MitoCarta 3.0 identified 28 MitoDEGs (Figure 1c).

Figure 1Identification of DEGs and MitoDEGs in the GSE38974 dataset. (a, b) Volcano plot and heatmap of DEGs between COPD and healthy control samples in the GSE38974 dataset. (c) Venn diagram showing the overlap between the DEGs and MitoCarta 3.0 database, identifying 28 MitoDEGs for further analysis.

**Figure:**
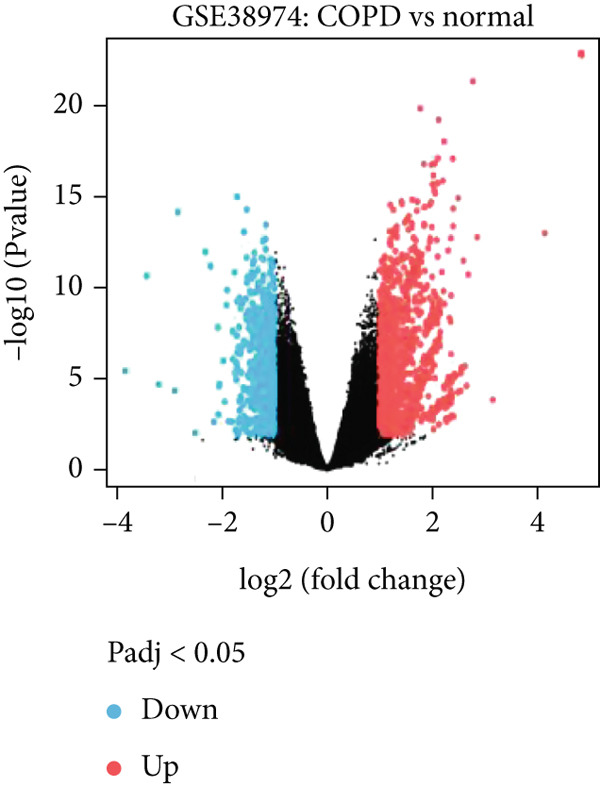
(a)

**Figure:**
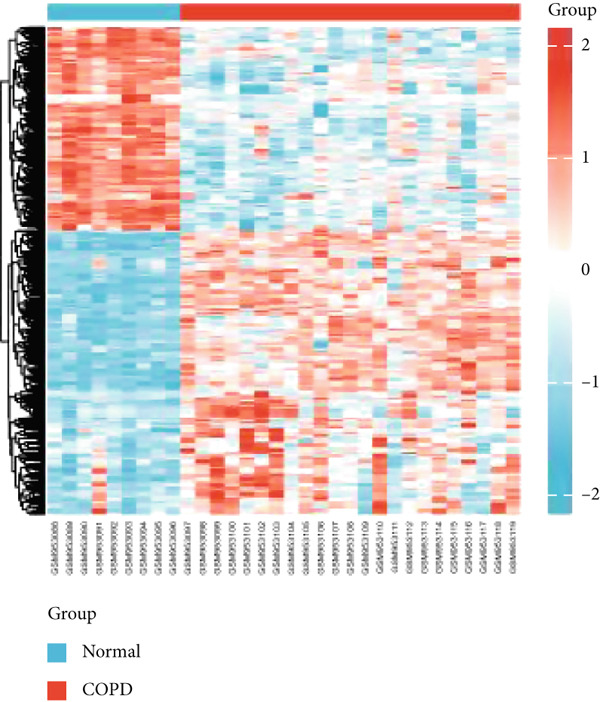
(b)

**Figure:**
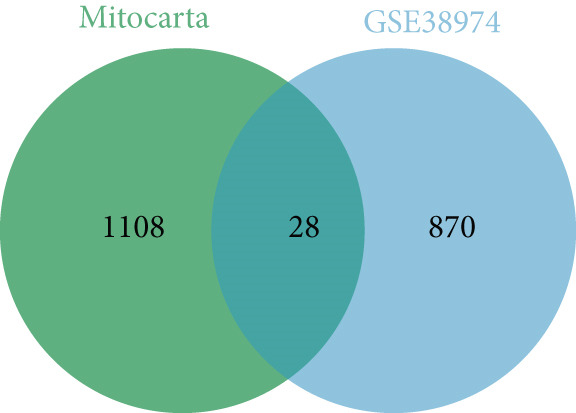
(c)

### 3.2. Functional Enrichment Analysis

Based on the 28 MitoDEGs, GO enrichment analysis revealed that these genes were predominantly associated with mitochondrial transport, regulation of cysteine‐type endopeptidase activity, and mitochondrial apoptosis in the BP category (Figure 2a,d). In terms of CC, there was a significant increase in mitochondrial DEGs in the extracellular membrane, organelle outer membrane, extracellular membrane, mitochondrial endometrium, and mitochondrial matrix (Figure 2b,e). The most highly enriched MF items included BH domain binding, electron transfer activity, death domain binding, and transferase activity (Figure 2c,f). KEGG pathway analysis revealed that mitochondrial DEGs were primarily correlated with apoptosis, platinum drug resistance, p53 signaling pathway, and lipid metabolism/atherosclerosis‐related pathways (Figure 3a,b).

Figure 2GO enrichment analysis of 28 MitoDEGs. (a–c) Bubble plots showing the significantly enriched GO terms in biological process (BP), cellular component (CC), and molecular function (MF). (d–f) Chord plots showing the significantly enriched GO terms in biological process (BP), cellular component (CC), and molecular function (MF).

**Figure:**
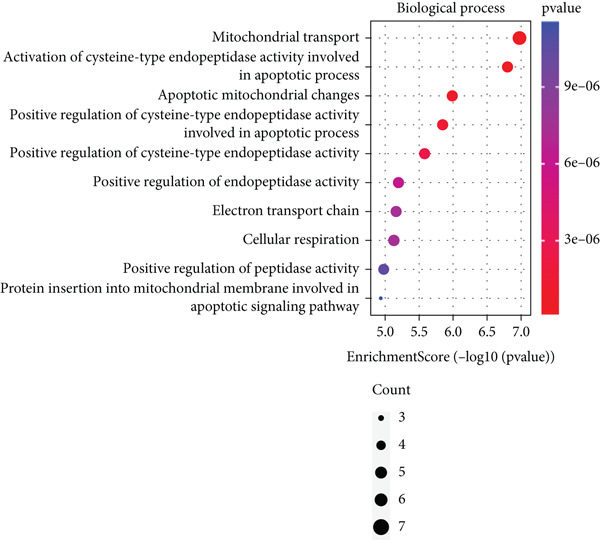
(a)

**Figure:**
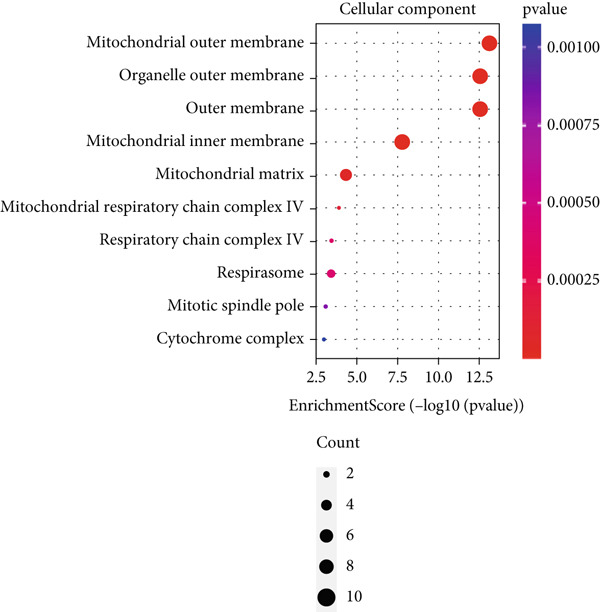
(b)

**Figure:**
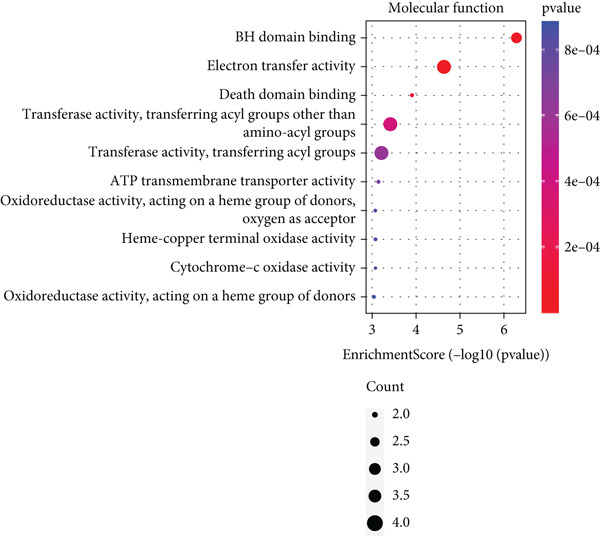
(c)

**Figure:**
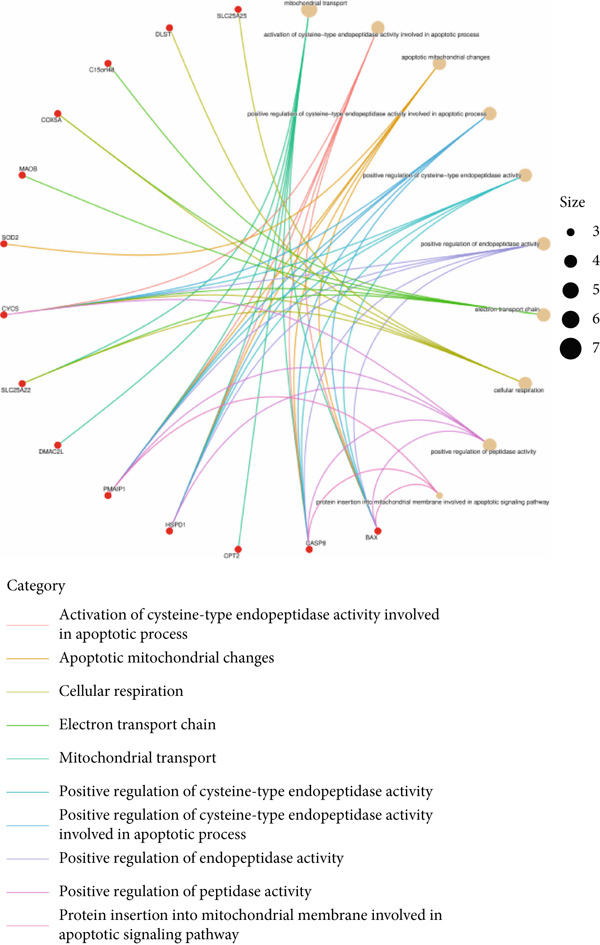
(d)

**Figure:**
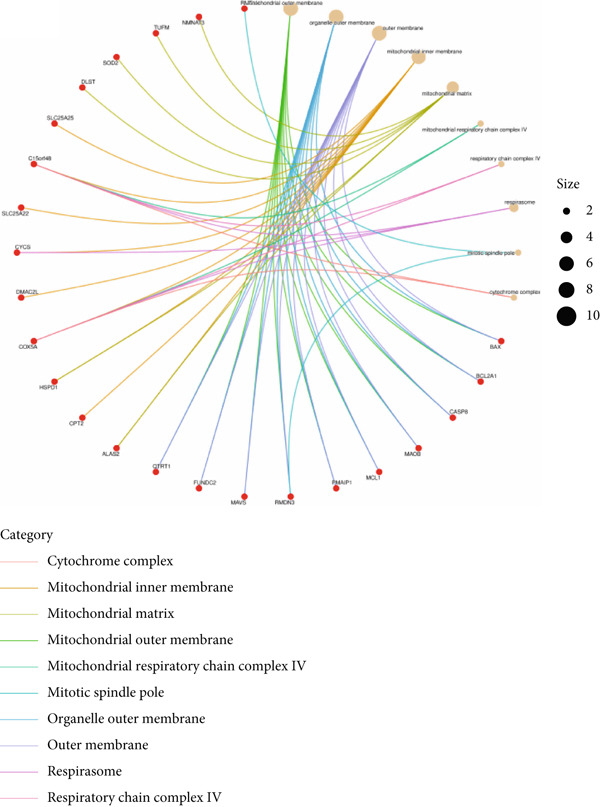
(e)

**Figure:**
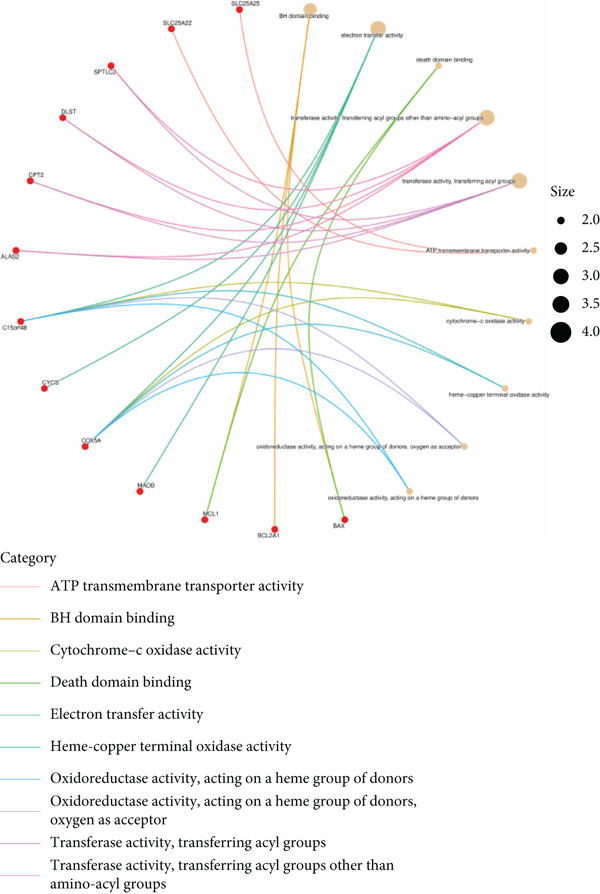
(f)

Figure 3KEGG pathway enrichment analysis of 28 MitoDEGs. (a) Bubble plot of significantly enriched KEGG pathways. (b) Chord plot of significantly enriched KEGG pathways.

**Figure:**
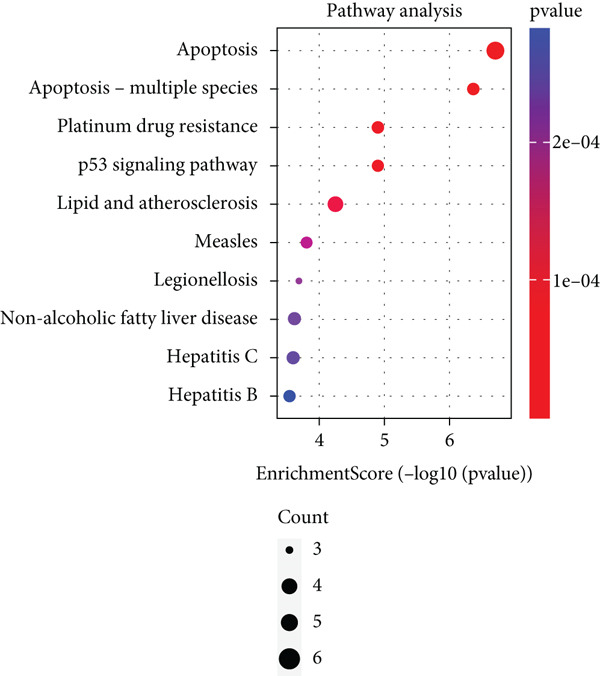
(a)

**Figure:**
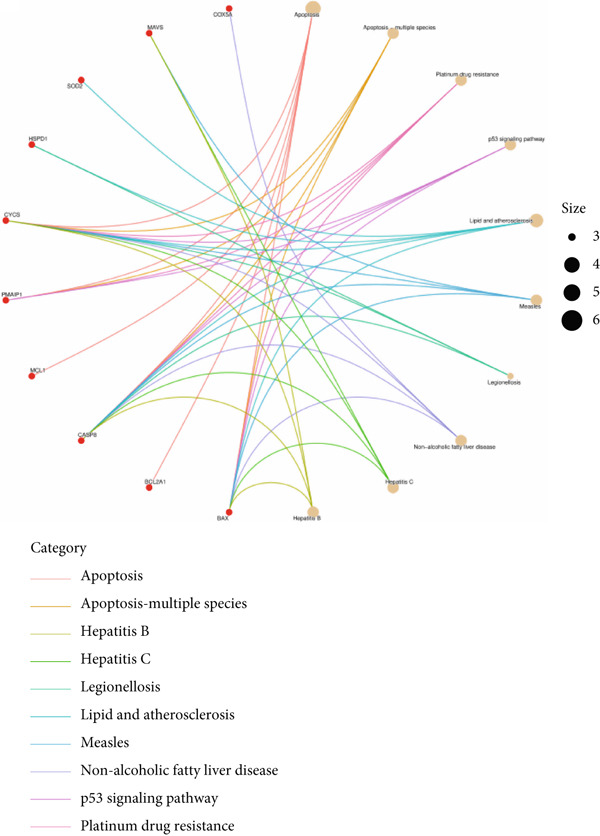
(b)

### 3.3. Feature Genes Identified Through Machine Learning

Applying LASSO regression with 10‐fold cross‐validation to the 28 MitoDEGs in GSE38974 yielded six nonzero‐coefficient genes: *BAX*, *DLST*, *FKBP10*, *FUNDC2*, *RMDN1*, and *COX5A* (Figure 4a,b). SVM‐RFE under 10‐fold cross‐validation identified 11 optimal features—*BAX*, *DLST*, *FKBP10*, *FUNDC2*, *RMDN1*, *MAOB*, *DMAC2L*, *CPT2*, *QTRT1*, *DUT*, and *SLC25A22* (Figure 4c,d). Considering the intersection of the outputs of the two algorithms, this study identified five primary features of MitoDEGs (*BAX*, *DLST*, *FKBP10*, *FUNDC2*, and *RMDN1*) (Figure 4e).

Figure 4Identification of feature genes using two machine learning algorithms. (a, b) Regression coefficient path diagram and cross‐validation curves for the LASSO logistic regression algorithm. (c, d) Change curve of accuracy and error rate of each gene in the SVM‐RFE algorithm. (e) Venn diagram showing the intersection of the diagnostic markers obtained from the two algorithms.

**Figure:**
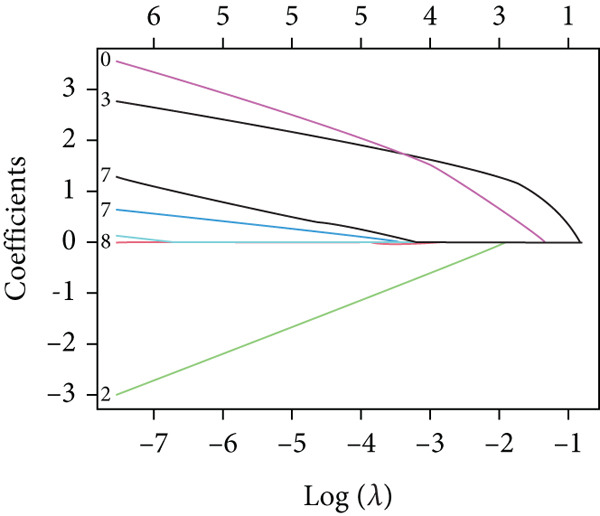
(a)

**Figure:**
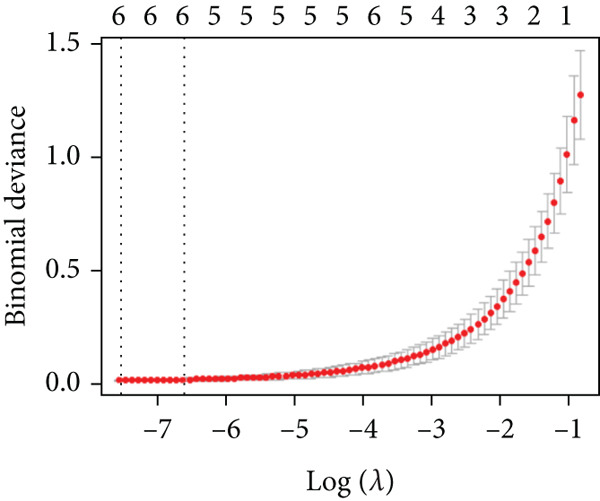
(b)

**Figure:**
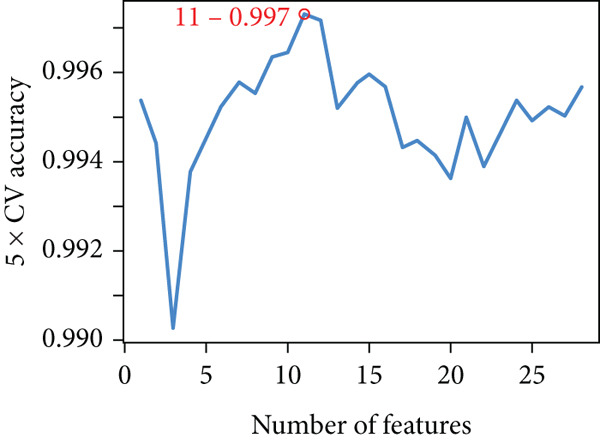
(c)

**Figure:**
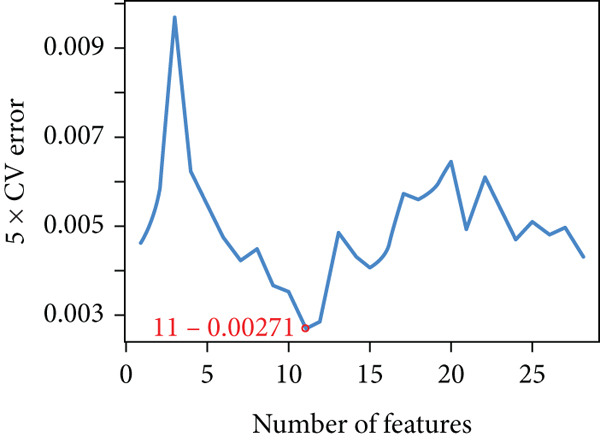
(d)

**Figure:**
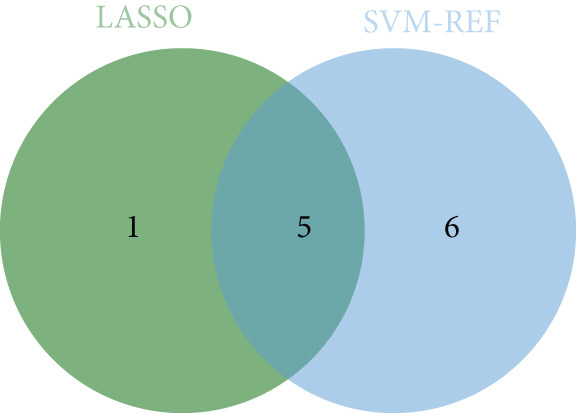
(e)

### 3.4. Verification of the Feature Genes in Training Set and Validation Set

To confirm the efficacy of feature MitoDEGs in COPD diagnosis, violin diagrams were generated to illustrate the gene expression in both the training set GSE38974 (Figure 5) and the verification set GSE8545 (Figure 6). It can be observed that COPD patients exhibit elevated levels of BAX and DLST expression compared to both training and validation normal control groups, with a statistically notable disparity. Nonetheless, there was a distinctive expression of FKBP10 and RMDN1 between COPD sufferers and healthy individuals in both the training and verification groups. Despite high levels of FUNDC2 expression in COPD patients across both groups, the validation group showed no significant variance, likely reflecting platform and cohort heterogeneity. Consequently, *BAX* and *DLST* were pinpointed as key genes for subsequent phases.

Figure 5Validation of the expression and diagnostic values of feature genes in the GSE38974 dataset. (a–e) Differences in the expression of BAX, DLST, FKBP10, FUNDC2, and RMDN1 between the COPD and control groups.  ^∗∗^ *p* < 0.001.

**Figure:**
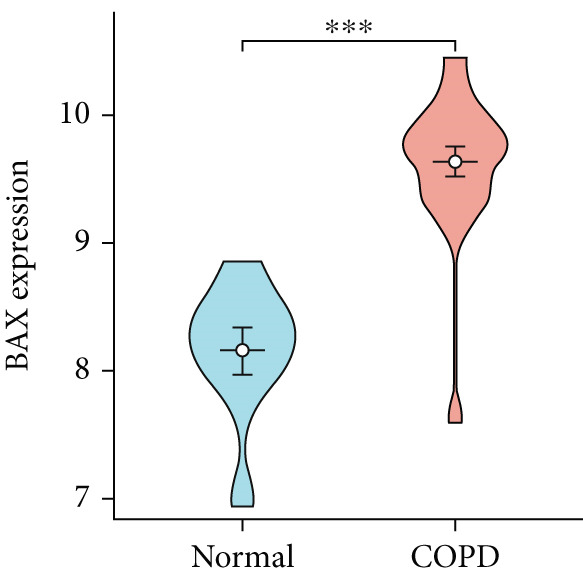
(a)

**Figure:**
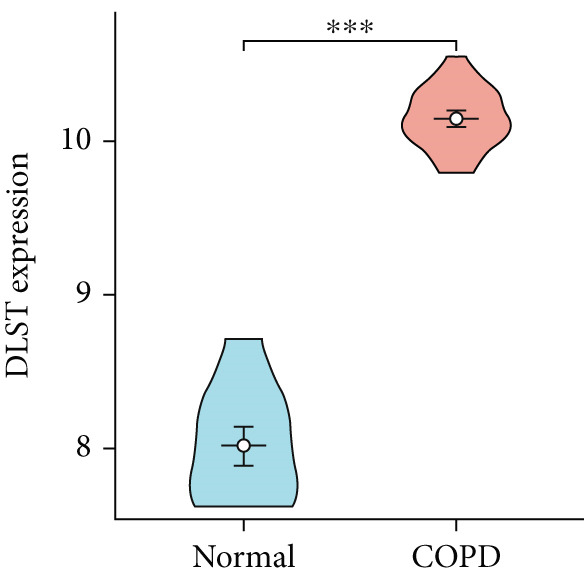
(b)

**Figure:**
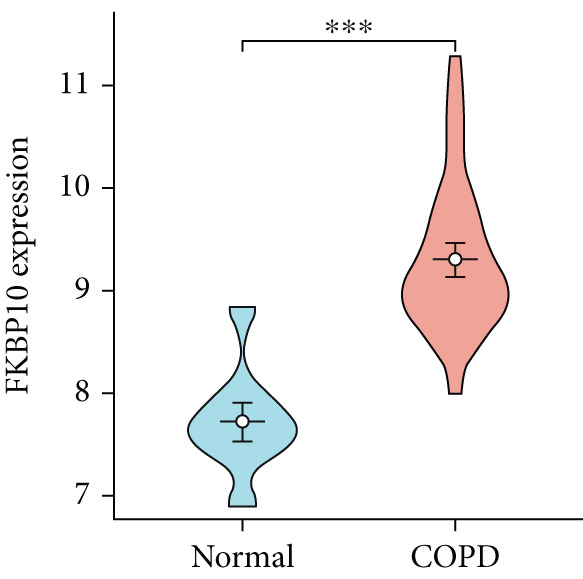
(c)

**Figure:**
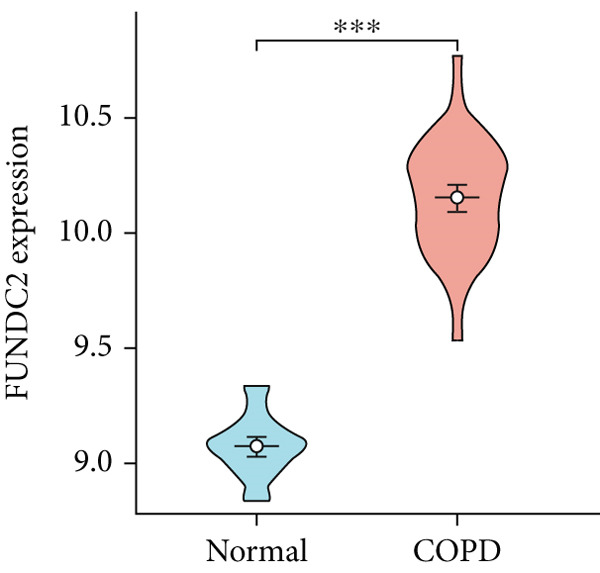
(d)

**Figure:**
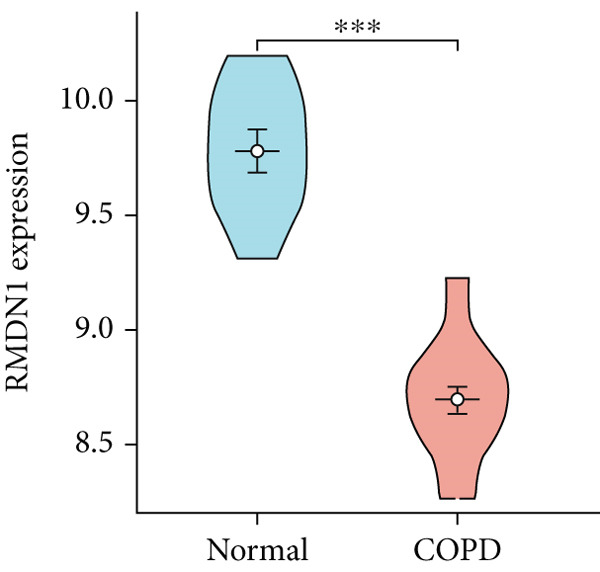
(e)

Figure 6Validation of the expression and diagnostic values of feature genes in the GSE8545 dataset. (a–e) Differences in the expression of BAX, DLST, FKBP10, FUNDC2, and RMDN1 between the COPD and control groups.  ^∗^ *p* < 0.05 and  ^∗∗^ *p* < 0.01.

**Figure:**
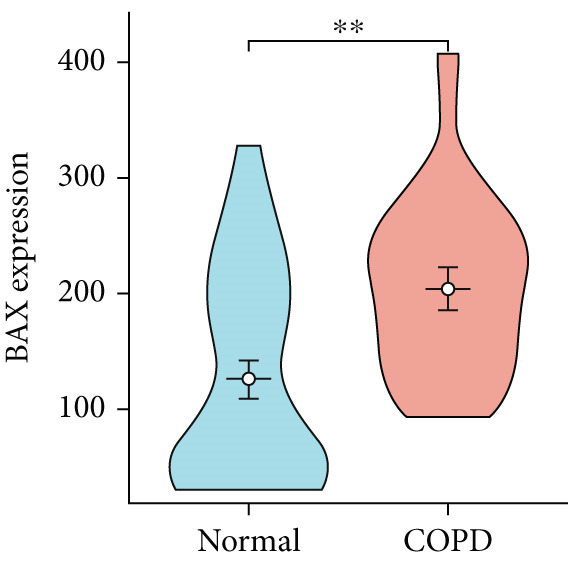
(a)

**Figure:**
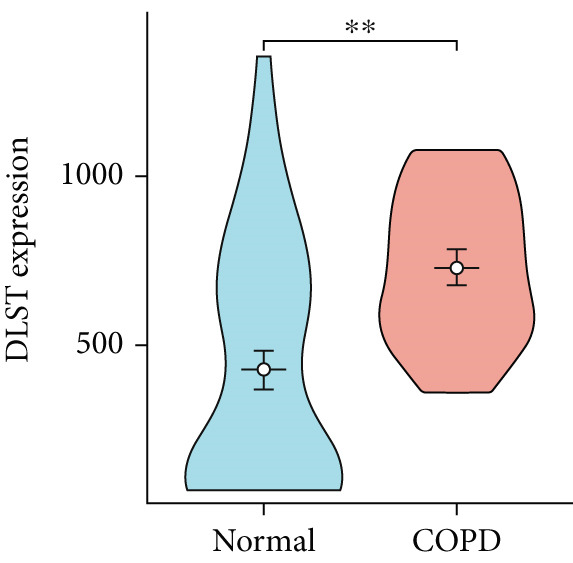
(b)

**Figure:**
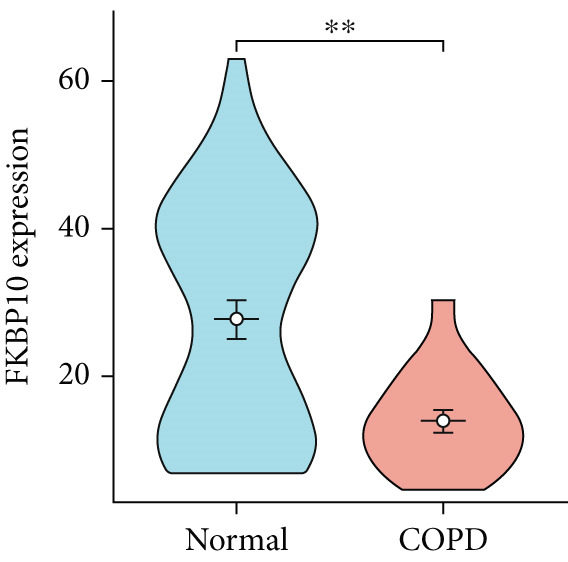
(c)

**Figure:**
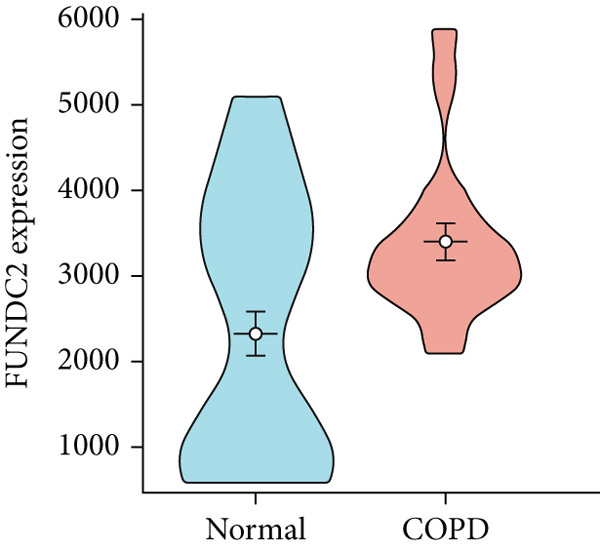
(d)

**Figure:**
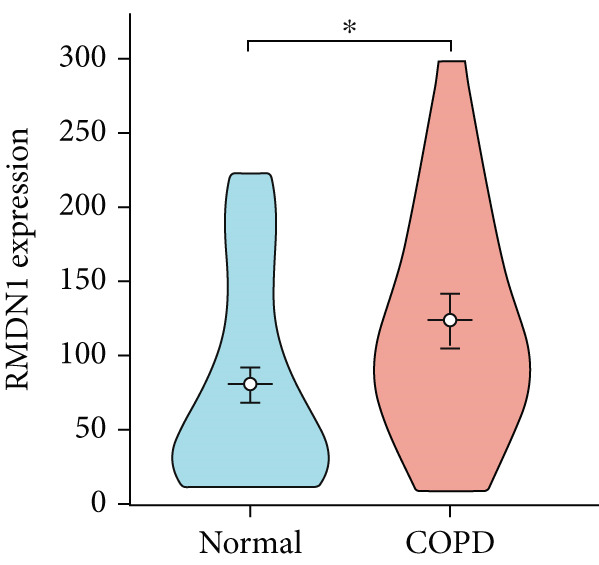
(e)

To investigate the central genes *BAX* and *DLST*, ROC curves were developed to evaluate their diagnostic significance in COPD. Within the training dataset, the AUC figures reached 0.961 for *BAX* and 0.976 for *DLST*, signifying superior diagnostic efficacy. Within the validation group, the AUC figures reached 0.742 for *BAX* and 0.741 for *DLST*, indicating a moderate level of diagnostic worth (Figure 7). Therefore, *BAX* and *DLST* excelled in COPD diagnosis, serving as potential markers to differentiate COPD specimens from standard samples.

Figure 7Diagnostic performance of BAX and DLST. ROC curves demonstrating the diagnostic efficacy of BAX and DLST in the (a, b) GSE38974 and (c, d) GSE8545 datasets. The AUC values are displayed for each gene.

**Figure:**
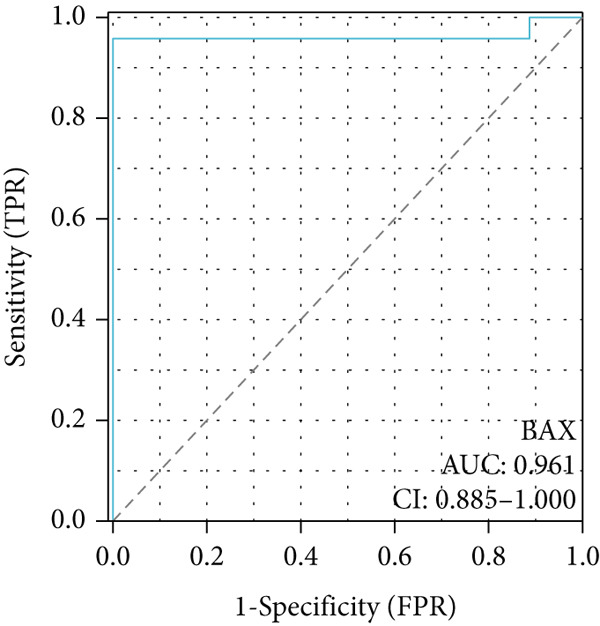
(a)

**Figure:**
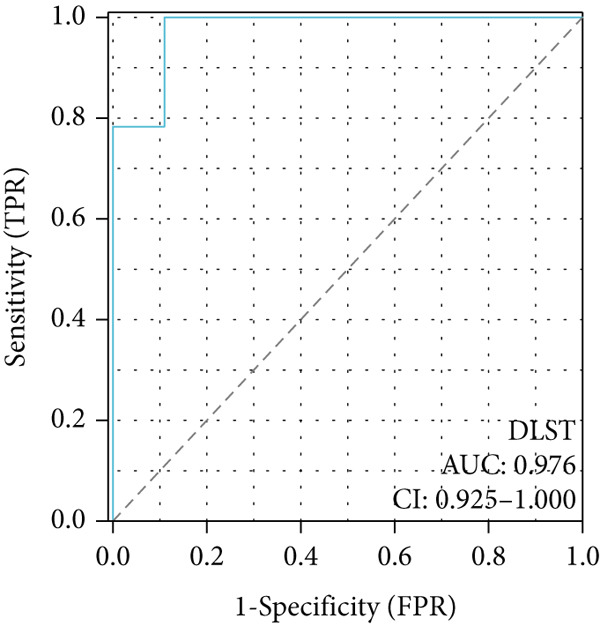
(b)

**Figure:**
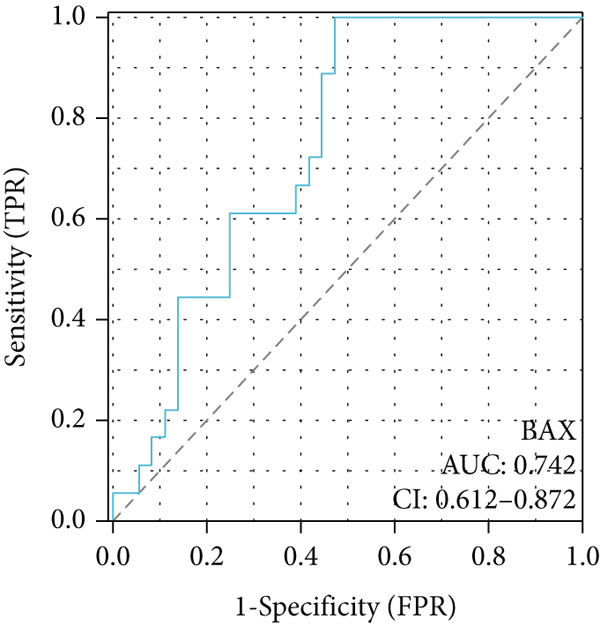
(c)

**Figure:**
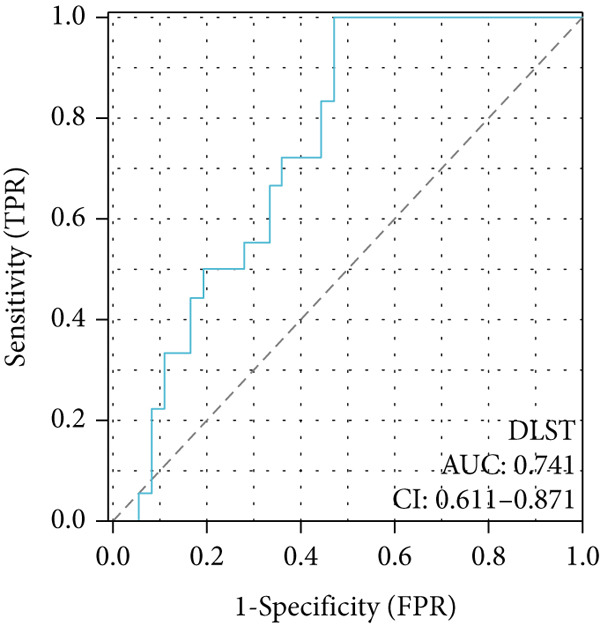
(d)

### 3.5. Verification of the Hub Genes In Vitro

Further analysis of hub MitoDEGs′ expression in bronchial epithelial cells (Beas‐2B and 16HBE) was conducted using qRT‐PCR after being subjected to different levels (0%–7%) of CSE. Figures 8a, 8b, 8c, and 8d illustrate that CSE‐treated cells exhibited notably elevated mRNA levels of BAX and DLST compared to unstimulated controls (*p* < 0.05), with a dose‐responsive rise in expression correlating to increased CSE concentrations. Consistent with the above results, Western blot analysis revealed a progressive increase in BAX and DLST protein concentrations correlating with higher CSE concentrations (*p* < 0.05) (Figures 8e, 8f, 8g, 8h, 8i, and 8j).

Figure 8CSE induces dose‐dependent upregulation of BAX and DLST in human bronchial epithelial cell lines (Beas‐2B and 16HBE cells). (a, b) BAX and DLST mRNA expression in Beas‐2B cells stimulated with 0%–7% CSE. (c, d) BAX and DLST mRNA expression in 16HBE cells stimulated with 0%–7% CSE. (e–g) BAX and DLST protein expression in Beas‐2B cells stimulated with 0%–7% CSE. (h–j) BAX and DLST protein expression in 16HBE cells stimulated with 0%–7% CSE.  ^∗^ *p* < 0.05,  ^∗∗^ *p* < 0.01,  ^∗∗∗^ *p* < 0.001, and  ^∗∗∗∗^ *p* < 0.0001.

**Figure:**
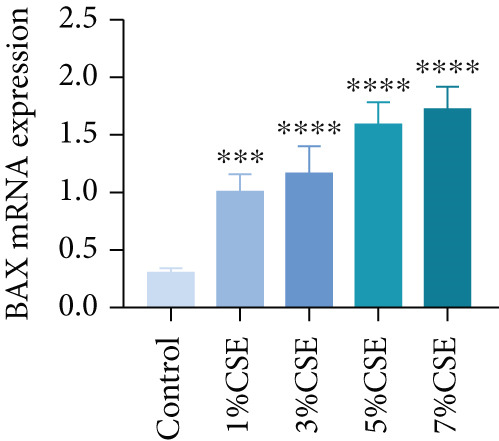
(a)

**Figure:**
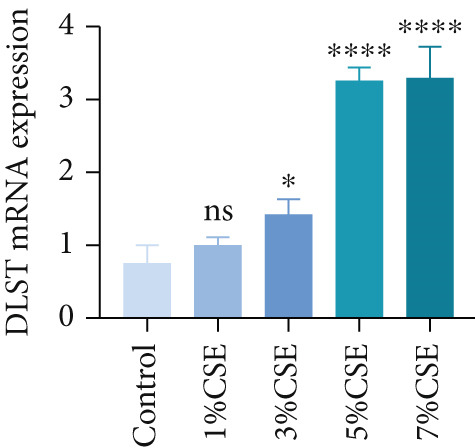
(b)

**Figure:**
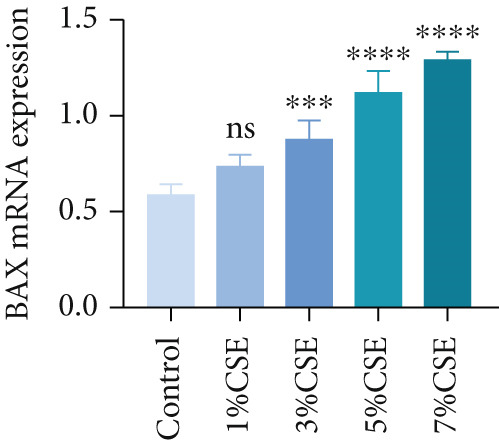
(c)

**Figure:**
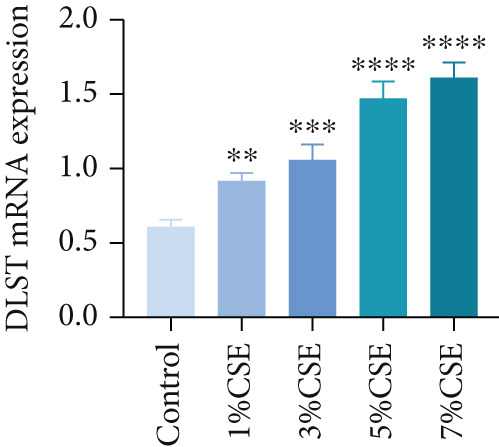
(d)

**Figure:**
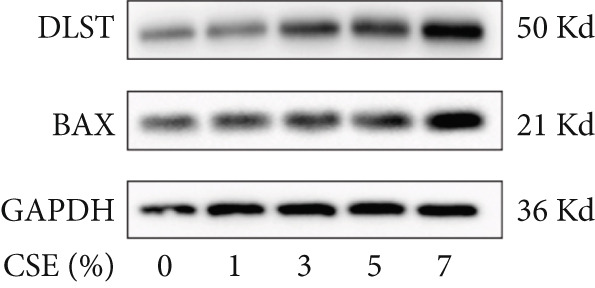
(e)

**Figure:**
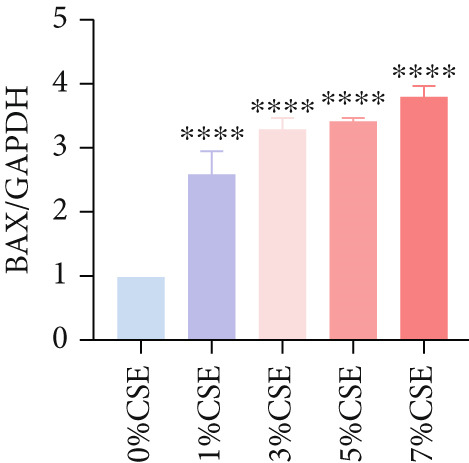
(f)

**Figure:**
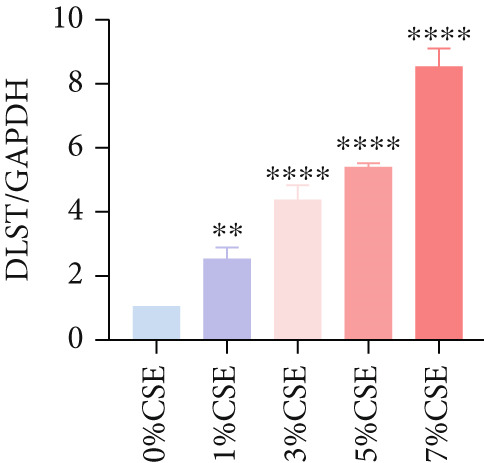
(g)

**Figure:**
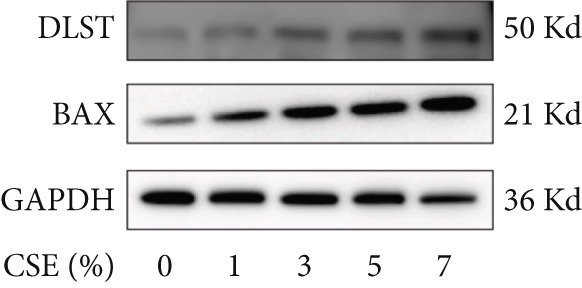
(h)

**Figure:**
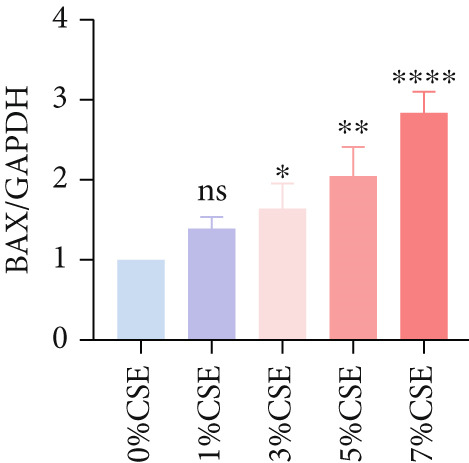
(i)

**Figure:**
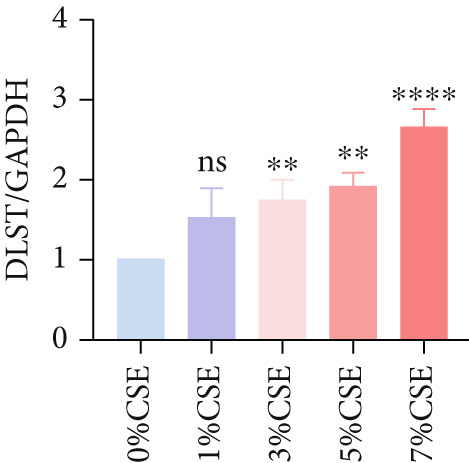
(j)

### 3.6. Verification of the Hub Genes In Vivo

To validate the in vitro findings, this study developed a CS‐induced COPD mouse model. Histopathological examination of H&E‐stained lung tissues from 6‐month smoke‐exposed mice exhibited characteristic COPD features, including alveolar enlargement and airway wall thickening, compared to control animals (Figure 9a). Quantitative analysis of alveolar damage showed that the MLI and DI were significantly increased in CS‐exposed mice, indicating pronounced emphysema changes (Figure 9b,c). Mouse lung tissues were homogenized and analyzed through Western blot, revealing increased expression of BAX and DLST in the lung tissues of CS‐exposed mice compared to controls (*p* < 0.05) (Figures 9d, 9e, and 9f).

Figure 9CS exposure induces emphysematous changes and upregulates BAX/DLST in mouse lung tissues. (a) Representative H&E‐stained lung sections from mice exposed to air or CS. Scale bar: 50 *μ*m. (b, c) Quantitative assessment of alveolar damage showing MLI and DI in air‐exposed and CS‐exposed mice. (d–f) BAX and DLST protein expression levels in lung tissue homogenates from mice exposed to air or CS.  ^∗∗^ *p* < 0.01 and  ^∗∗∗∗^ *p* < 0.0001.

**Figure:**
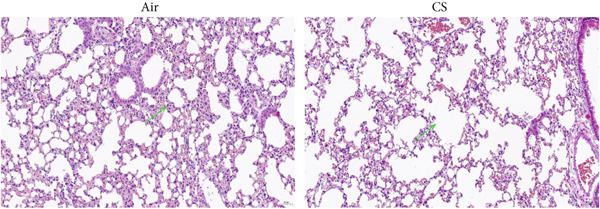
(a)

**Figure:**
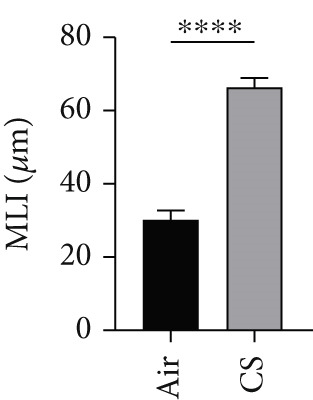
(b)

**Figure:**
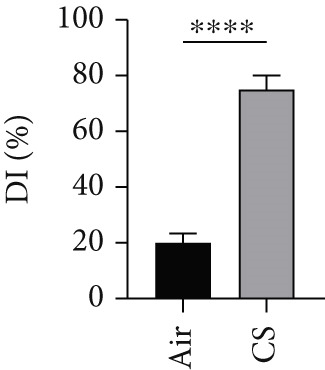
(c)

**Figure:**
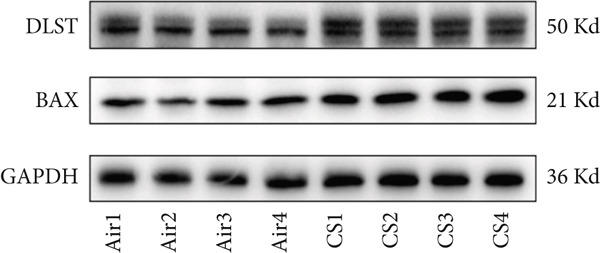
(d)

**Figure:**
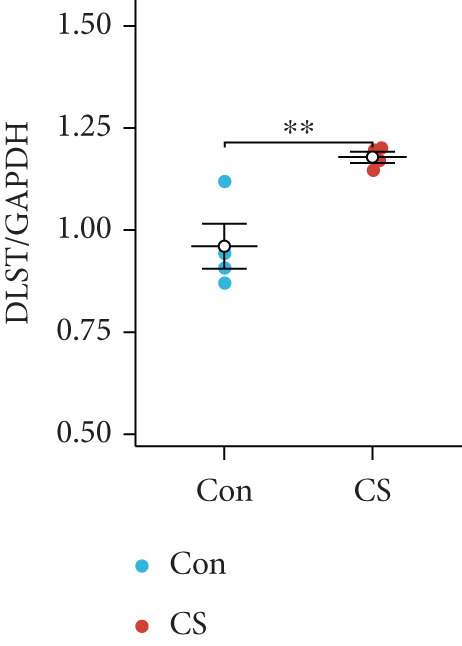
(e)

**Figure:**
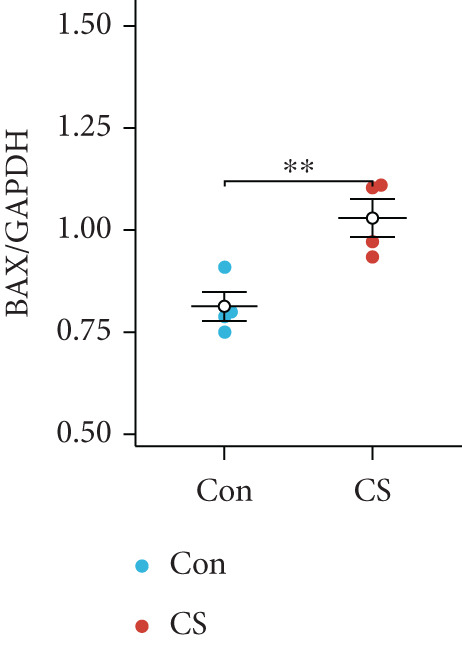
(f)

### 3.7. Immune Infiltration and Correlation Analysis

Utilizing CIBERSORT algorithms, this study evaluated the immune penetration of COPD, determined the proportion of immune cells, and graphed them in a layered histogram (Figure 10a). The correlation heatmap, which was created to assess the relationship among 22 varieties of immune cell infiltration (Figure 10b), indicated that macrophages M1 had notable positive associations with activated CD4 memory T cells, gamma delta T cells, and follicular helper T cells. Conversely, a significant inverse correlation was observed between macrophages M2 and monocytes and also between macrophages M0 and activated NK cells. A box plot (Figure 10c) was then crafted to explore the variances in the penetration of 22 types of immune cells. The boxplot illustrates that in COPD samples, macrophages M0 and monocytes exhibited greater infiltration than in control samples, whereas T cells CD8 and NK cells were activated, and macrophages M2 and resting mast cells showed lesser infiltration.

Figure 10CIBERSORT analysis of infiltrating immune cells in the COPD and control groups. (a) Stacked histogram showing the distribution of 22 immune cells in COPD and control groups. (b) Correlation heatmap of 22 immune cell types. The positive and negative correlations are shown in red and blue, respectively. (c) Violin diagram showing differences in immune cell infiltration between the COPD and control groups.

**Figure:**
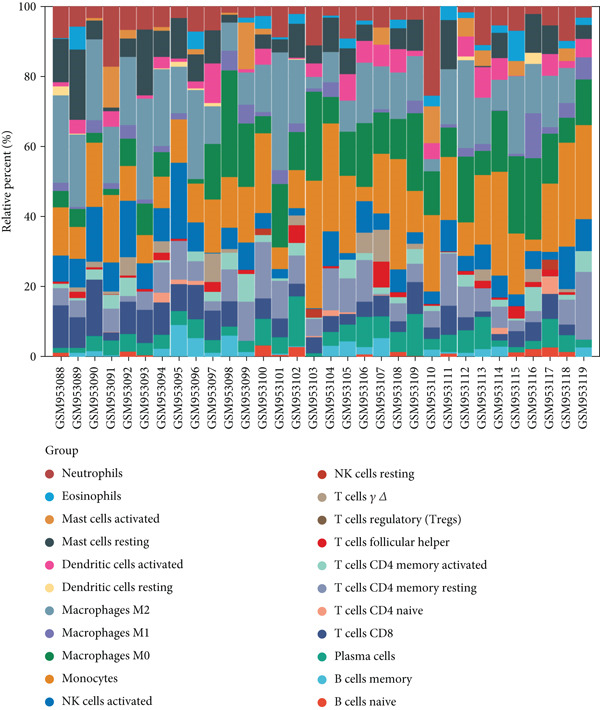
(a)

**Figure:**
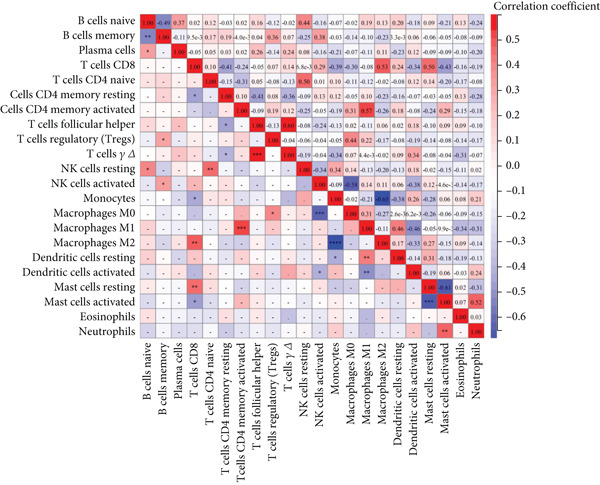
(b)

**Figure:**
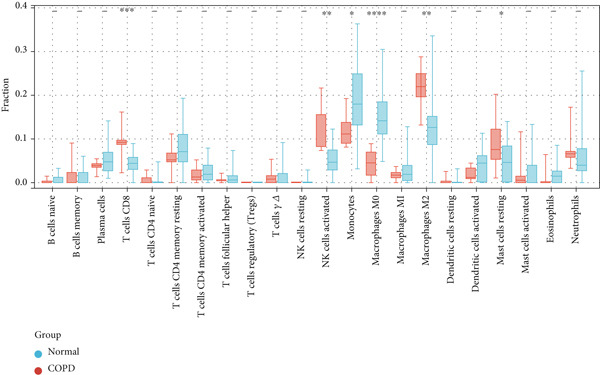
(c)

To further validate these computational findings, we performed IHC on mouse lung tissues exposed to air or CS using representative immune markers including F4/80 (macrophages M0), CD206 (macrophages M2), Ly6C (monocytes), and CD8a (T cells CD8) (Figure 11a). The IHC results consistently supported our earlier observations. Specifically, we observed a significant increase in F4/80^+^ M0 macrophages (Figure 11b) and Ly6C^+^ monocytes (Figure 11c) in CS‐exposed mouse lung tissues compared to air‐exposed controls. In contrast, a marked decrease was detected in CD8a^+^ T cells (Figure 11d) and CD206^+^ M2 macrophages (Figure 11e). These findings align well with the CIBERSORT data, which indicated elevated infiltration of M0 macrophages and monocytes, and reduced infiltration of M2 macrophages and CD8^+^ T cells in COPD samples. This experimental validation strengthens the reliability of our immune infiltration analysis and confirms the alterations in key immune cell populations within the COPD microenvironment.

Figure 11IHC validation of immune cell infiltration in CS‐exposed mouse lungs. (a) Representative IHC staining images of F4/80 (macrophages M0), Ly6C (monocytes), CD8a (T cells CD8), and CD206 (macrophages M2) in lung tissues from air‐exposed (air) and CS‐exposed (CS) mice. Right panels show higher magnification views of the boxed regions. Quantification of the percentage of positively stained area for (b) F4/80, (c) Ly6C, (d) CD8a, and (e) CD206 in lung sections from air and CS groups.  ^∗∗∗^ *p* < 0.001 and  ^∗∗∗∗^ *p* < 0.0001.

**Figure:**
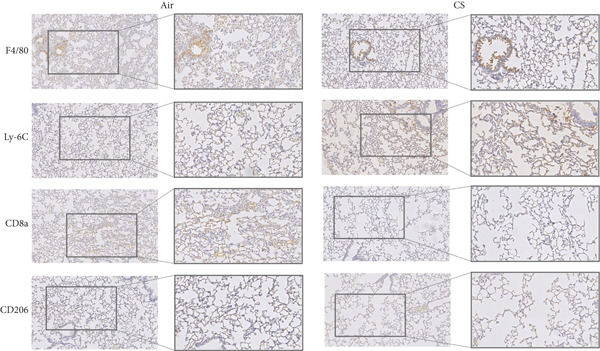
(a)

**Figure:**
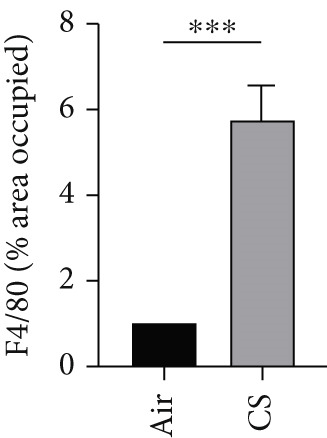
(b)

**Figure:**
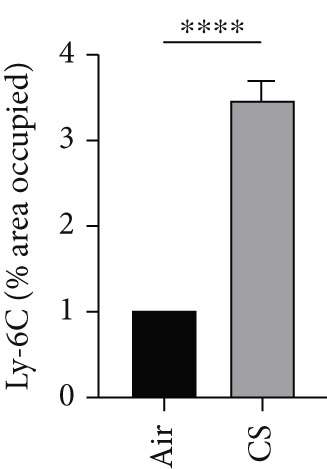
(c)

**Figure:**
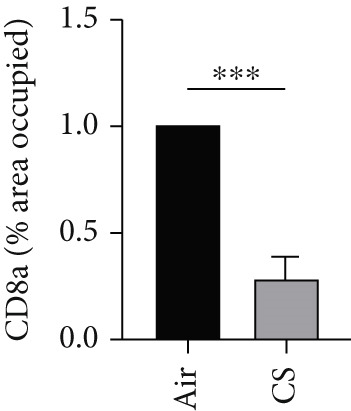
(d)

**Figure:**
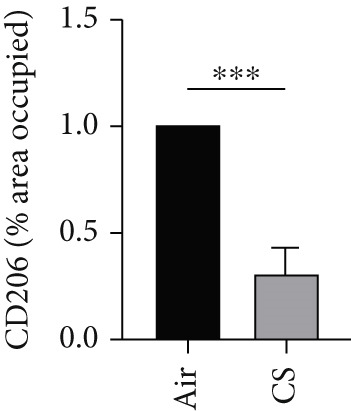
(e)

### 3.8. Correlation Analysis Between Hub Genes and Infiltrating Immune Cells

There was a notable positive link between BAX expression and M0 macrophages (*p* = 0.007) as well as naive B cells (*p* = 0.046) in contrast to a significant negative association with activated NK cells (*p* = 0.002) (Figure 12a). The expression of DLST showed a robust positive link with M0 macrophages (*p* < 0.001) and dormant NK cells (*p* = 0.034), yet a notable negative correlation with activated NK cells (*p* = 0.005), neutrophils (*p* = 0.020), M2 macrophages (*p* = 0.022), and CD8^+^ T cells (*p* = 0.033) (Figure 12b).

Figure 12Correlation analysis between hub gene expression and immune cell infiltration. Lollipop plots showing the correlation between (a) BAX or (b) DLST expression and immune cells.

**Figure:**
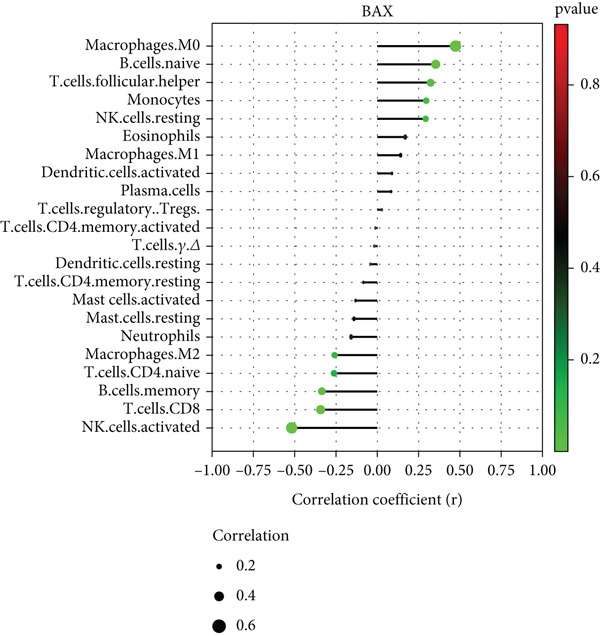
(a)

**Figure:**
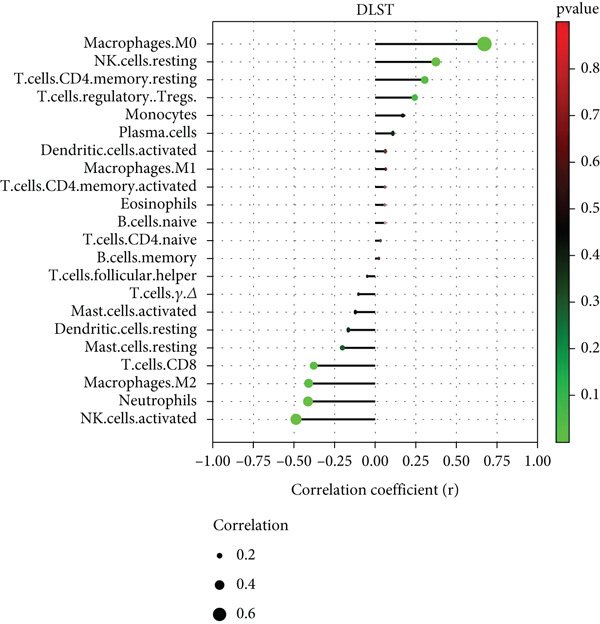
(b)

To experimentally validate these computational findings, we performed IF on lung tissue sections from CS‐exposed and air‐exposed mice. Specifically, BAX or DLST was costained with F4/80 (a marker of macrophages M0) and with NKp46 together with GranzymeB (markers of activated NK cells). Quantitative image analysis demonstrated that epithelial BAX and DLST expression levels were positively correlated with the density of macrophages (F4/80^+^) (Figure 13) and negatively correlated with the density of activated NK cells (NKp46^+^/GranzymeB^+^) (Figure 14), thereby supporting the computational correlation analysis.

Figure 13IF costaining of BAX/DLST with F4/80 to assess correlations of hub genes with macrophages M0 in mouse lung tissues. (a) Representative IF images and quantification of the colocalization between BAX and F4/80 in lung sections from air‐exposed (air) and CS‐exposed (CS) mice. (b) Representative IF images and quantification of the colocalization between DLST and F4/80 in lung sections from Air and CS mice. Nuclei were counterstained with DAPI.  ^∗∗^ *p* < 0.01 and  ^∗∗∗∗^ *p* < 0.001.

**Figure:**
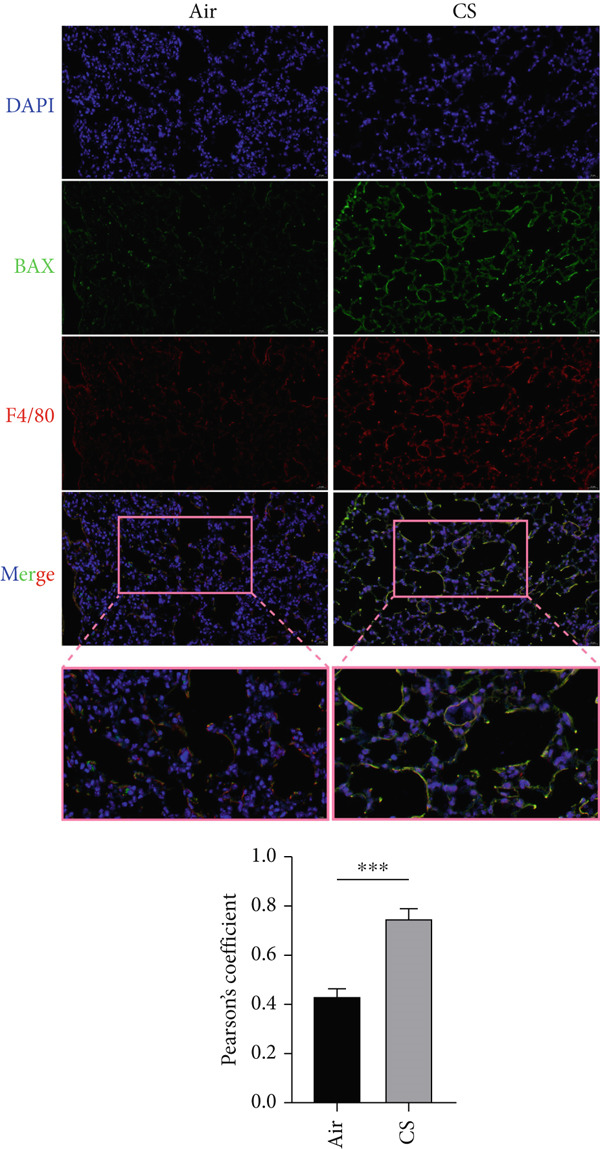
(a)

**Figure:**
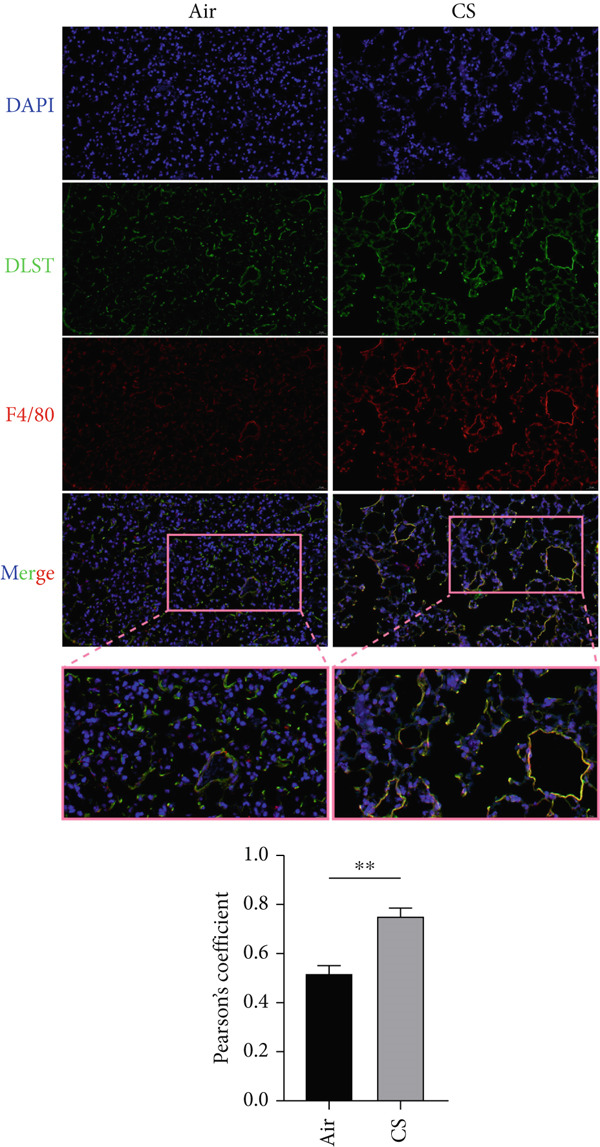
(b)

Figure 14IF costaining of BAX/DLST with NKp46 and GranzymeB to assess correlations of hub genes with activated NK cells in mouse lung tissues. (a) Representative IF images and quantification of the colocalization between BAX, NKp46, and GranzymeB in lung sections from air and CS mice. (b) Representative IF images and quantification of the colocalization between DLST, NKp46, and GranzymeB in lung sections from air and CS mice. Nuclei were counterstained with DAPI.  ^∗∗^ *p* < 0.01 and  ^∗∗∗^ *p* < 0.001.

**Figure:**
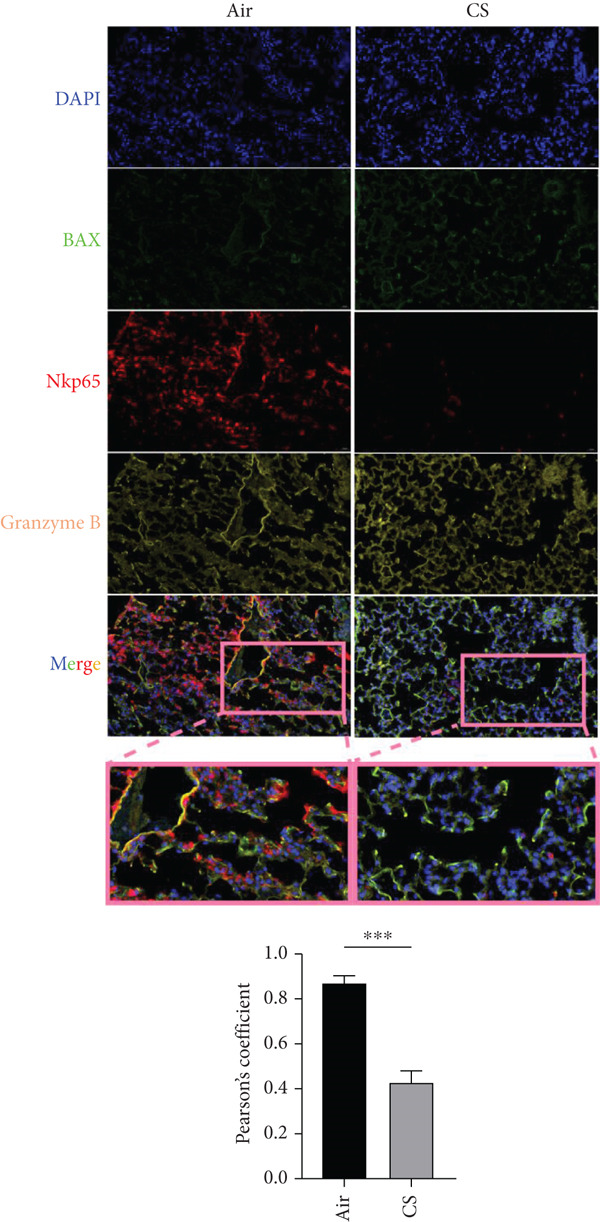
(a)

**Figure:**
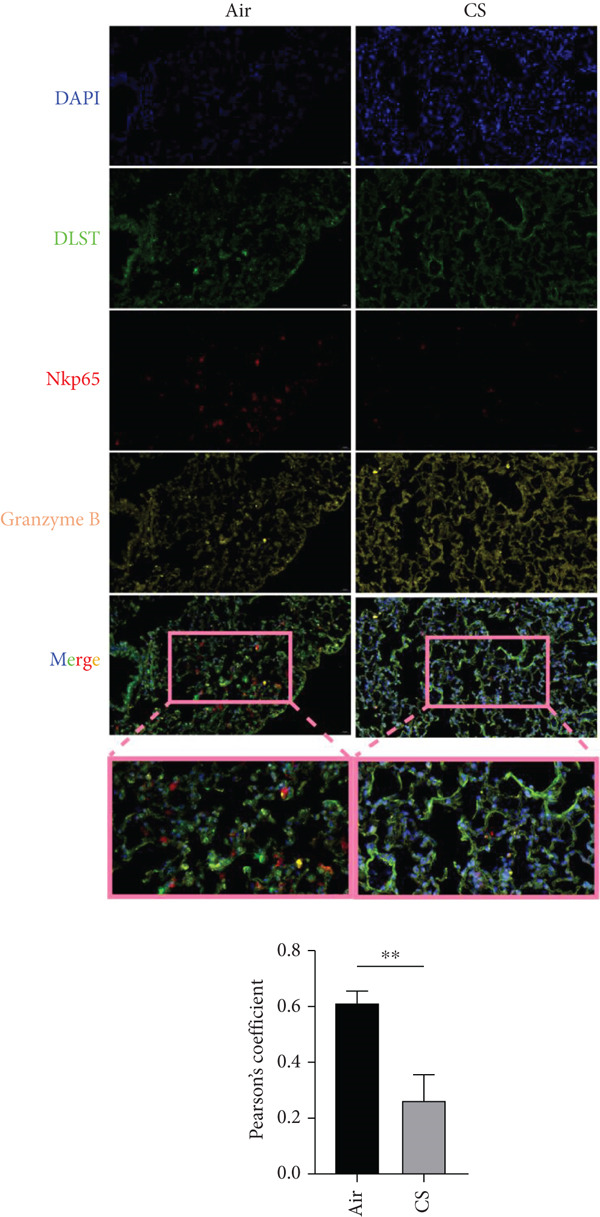
(b)

## 4. Discussion

Despite advancements in COPD diagnosis and treatment, prognosis remains poor due to an incomplete understanding of its pathogenesis [11]. Further mechanistic insights are essential for identifying novel therapeutic targets [12]. This study integrated the airway epithelial cell dataset GSE38974 with the mitochondrial gene set from Mitocarta 3.0, identifying 28 MitoDEGs. Using LASSO regression and SVM‐RFE, five characteristic genes were screened, and two hub genes—BAX and DLST—were validated via qRT‐PCR, Western blot, and the GSE8545 dataset. Considering the immune system′s role in COPD [13, 14], we also explored immune infiltration using CIBERSORT.

*BAX*, a proapoptotic gene in the Bcl‐2 family, regulates mitochondrial‐mediated apoptosis. Its interaction with Bcl‐2 and its expression level relative to Bcl‐2 (the BAX/Bcl‐2 ratio) critically determines apoptotic susceptibility [15–17]. Apoptosis is a recognized contributor to COPD, with increased apoptotic epithelial and endothelial cells documented in COPD lungs, leading to irreversible tissue damage and emphysema [18–21]. Prior studies have confirmed elevated BAX expression in COPD [22]. However, inconsistencies remain, as apoptosis is not universally observed in emphysematous tissues, and smoking can paradoxically promote or suppress apoptosis [23, 24]. These complexities highlight the need for further investigation into apoptosis‐mediated mechanisms in COPD.

*DLST*, encoding dihydrolipoamide S‐succinyltransferase, is a core component of the *α*‐ketoglutarate dehydrogenase complex in the TCA cycle [25]. It facilitates the conversion of *α*‐ketoglutarate to succinyl‐CoA, linking carbohydrate, lipid, and amino acid metabolism [26]. DLST dysregulation has been implicated in several conditions, including cardiac dysfunction, neurodegeneration, and malignancies [27–31]. In COPD, we observed increased DLST expression in airway epithelial cells, supported by in vitro validation. Intracellular copper accumulation can disrupt TCA cycle activity via protein acylation, triggering cuproptosis [32, 33]. Although serum copper is elevated in COPD patients [34], studies on cuproptosis in COPD are limited. Our findings suggest that DLST may contribute to COPD pathogenesis through mitochondrial metabolic stress, warranting further study.

Notably, FKBP10 and RMDN1 showed inconsistent expression trends between the GSE38974 and GSE8545 datasets. This discrepancy may arise from several factors. First, technical differences such as probe design sensitivity, normalization methods, and sequencing depth in microarray platforms (GPL433 vs. GPL570) may introduce systematic measurement bias. Second, cohort‐specific biological variability such as smoking status (e.g., current vs. former smokers), sampling site (e.g., airway vs. alveolar tissue), and disease severity (e.g., GOLD stages) could drive divergent gene expression patterns. Third, statistical overfitting during feature selection may also influence the results [35, 36]. Future studies using single‐cell RNA sequencing or larger, well‐annotated cohorts are needed to resolve such inconsistencies and improve biomarker reliability [37].

Immune infiltration analysis revealed increased macrophages M0 and monocytes in COPD samples, along with decreased T cells CD8, activated NK cells, macrophages M2, and resting mast cells. BAX and DLST expression were positively correlated with macrophages M0 and inversely correlated with activated NK cells. These bioinformatic findings were subsequently validated experimentally using IHC and IF in lung tissues from our CS‐induced COPD mouse model. These results align with prior studies reporting macrophage dominance and T cell reduction in COPD lungs [38]. Macrophages in COPD are key mediators of inflammation and phagocytosis and exhibit functional heterogeneity [39–41]. Elevated M0 macrophages may reflect increased polarization potential, while reduced M2 macrophages indicate compromised anti‐inflammatory capacity [42]. Reports on T cell CD8 levels remain contradictory [43–46], with both increased and decreased levels described in COPD patients and smokers, possibly reflecting methodological or population differences.

However, this study has limitations. First, the modest sample size (*n* = 71; 38 COPD and 33 controls) limits statistical power and the robustness of the results, particularly given the heterogeneity of COPD. Second, the datasets lacked detailed clinical metadata—such as COPD subtypes, severity scores, spirometric parameters (FEV_1_ and FVC), smoking history, age, and sex—limiting the generalizability and clinical relevance of the findings. Third, the binary classification approach (“COPD vs. healthy”) oversimplifies COPD′s complex phenotypic spectrum. Consequently, the diagnostic and mechanistic significance of BAX and DLST remains preliminary; future studies using larger, well‐characterized, and stratified cohorts with comprehensive clinical information are warranted to validate and extend these findings across diverse patient populations.

## 5. Conclusions

To sum up, *BAX* and *DLST* stand as promising biomarkers for diagnosing COPD and potential targets for treatment. Additionally, these molecular indicators show notable correlations with the infiltration patterns of immune cells, offering mechanistic guidance for upcoming approaches in the modulation of COPD pathogenesis and in clinical treatment.

## Conflicts of Interest

The authors declare no conflicts of interest.

## Funding

No funding was received for this manuscript.

## Data Availability

The datasets analyzed during the current study are publicly available from the Gene Expression Omnibus database: GSE38974 (https://www.ncbi.nlm.nih.gov/geo/query/acc.cgi?acc=GSE38974) and GSE8545 (https://www.ncbi.nlm.nih.gov/geo/query/acc.cgi?acc=GSE8545).
